# Sensing of RNA stress by mTORC1 drives autoinflammation

**DOI:** 10.1172/JCI156119

**Published:** 2022-01-18

**Authors:** Min Ae Lee-Kirsch

**Affiliations:** Department of Pediatrics, Medizinische Fakultät Carl Gustav Carus, Technische Universität Dresden, Dresden, Germany.

## Abstract

Loss-of-function mutations in *SKIV2L* underlie trichohepatoenteric syndrome (THES2), a rare inborn error of immunity characterized by diarrhea, skin lesions, brittle hair, and immunodeficiency. SKIV2L is part of a multiprotein complex required for exosome-mediated RNA surveillance through RNA decay. In this issue of the *JCI*, Yang et al. delineate a mechanism underlying autoinflammatory skin disease in *Skiv2l*-deficient mice. Thus, a lack of SKIV2L activates mTORC1 signaling in keratinocytes and T cells, impeding skin barrier integrity and T cell homeostasis. Interestingly, treatment with the mTOR inhibitor rapamycin improves skin symptoms in *Skiv2l*-deficient mice, suggesting a possible therapeutic avenue for patients with THES2.

## RNA metabolism and RNA sensing

**Figure 1:**
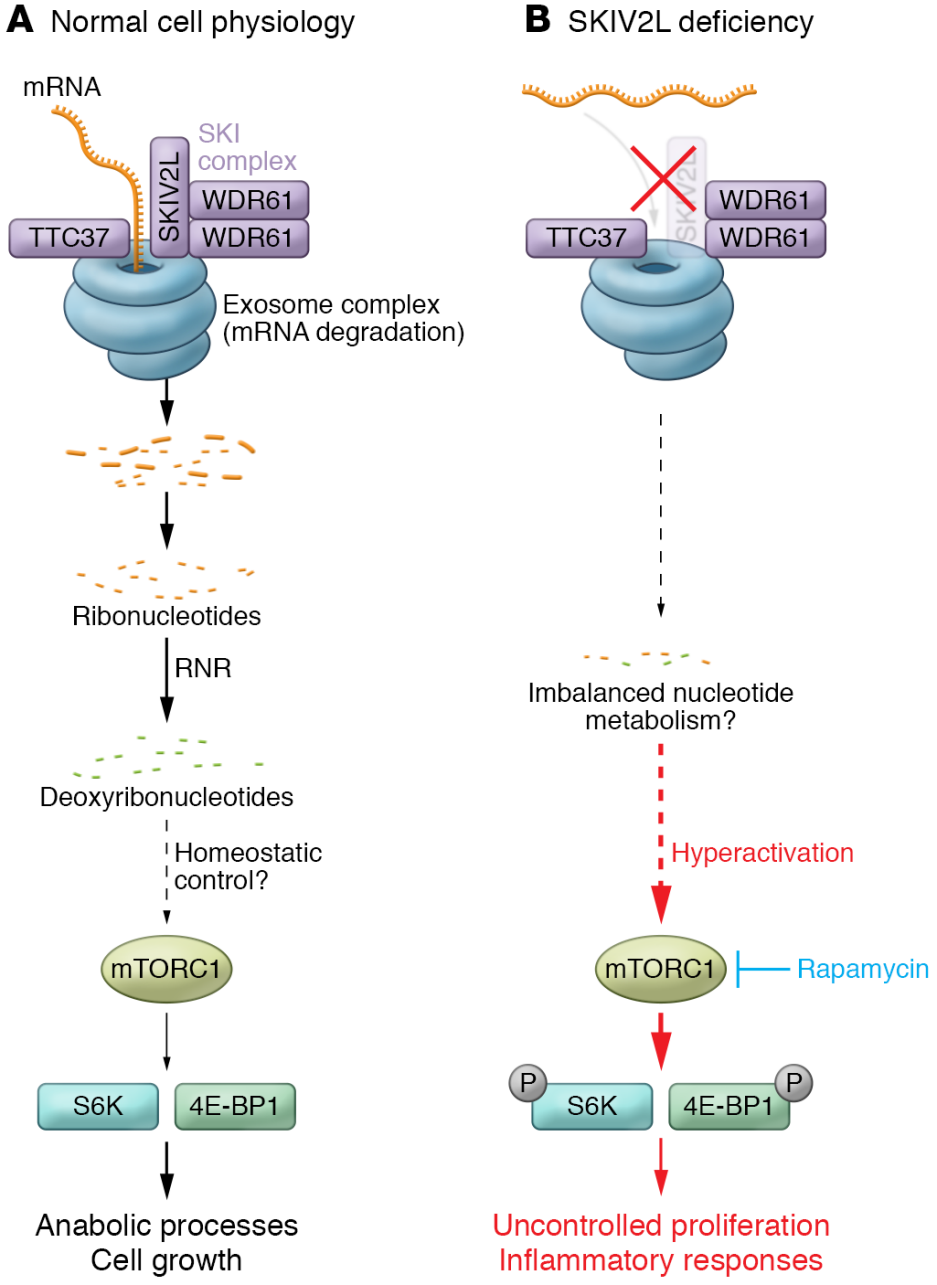
A model for mTORC1 signaling in SKIV2L deficiency that leads to uncontrolled proliferation and immune activation. (**A**) The turnover and quality control of ribosome-associated mRNA are controlled by the cytoplasmic RNA exosome, which acts as a molecular shredder by degrading mRNA from the 3′ end. The RNA exosome is assisted by the SKI complex. The SKIV2L helicase unwinds RNA and, together with the TPR motif–containing protein TTC37 and two subunits of the WD-repeat protein WDR61, initiates ribonucleolytic degradation. The mTORC1 complex senses nutrient deprivation, which induces activation of downstream targets S6K and 4E-BP1 by phosphorylation. mTORC1 signaling promotes a broad range of anabolic processes, including cell growth and proliferation, which require a sufficient supply of deoxyribonucleotides for DNA synthesis. (**B**) Aberrant mTORC1 signaling in SKIV2L deficiency may be triggered by sensing changes in cellular concentrations of deoxyribonucleotides, which are produced from ribonucleotides by the action of ribonucleotide reductase (RNR). Unabated mTORC1 activity in keratinocytes and T cells leads to hyperproliferation with secondary inflammatory responses. Rapamycin inhibits the protein kinase mTOR, core component of the mTORC1 complex, and may therefore be of therapeutic benefit in patients with SKIV2L deficiency.

The human genome is transcribed to generate an extraordinary diversity of RNA, which is subject to complex regulation to maintain cellular homeostasis. The turnover and quality control of ribosome-associated mRNA are controlled by the cytoplasmic RNA exosome, an RNA degradation machinery acting in concert with the super-killer (SKI) complex (1). The SKI complex consists of the SKIV2L helicase, two subunits of the WD-repeat protein WDR61, and the tetratricopeptide repeat motif containing protein TTC37 (2). It associates with the 80S ribosome and extracts mRNA that is no longer needed or recognized as being faulty to initiate ribonucleolytic degradation from the 3′ end by the RNA exosome (Figure 1) (3, 4).

RNA metabolism is also critical for immune homeostasis, as self-RNA occurring in the wrong place at the wrong time can turn into a danger signal that triggers immune responses leading to autoinflammation or autoimmunity (5). Sensing of viral RNA by receptors of the innate immune system is an essential strategy in antiviral immunity (6). A central challenge for the host cell is to discriminate between harmful foreign RNA and self-RNA. Ligand specificity of RNA sensors, such as the cytosolic RNA helicases RIG-I and MDA5, relies largely on unique structural properties of viral RNA (5, 6). Thus, while RIG-I senses 5′-triphosphate-RNA or 5′-diphosphate-RNA, MDA5 recognizes long, double-stranded RNA (7–9). Engagement of these RNA sensors triggers type I interferon (IFN) signaling, resulting in the activation of numerous antiviral transcriptional programs (5, 6). The processes of RNA metabolism and RNA sensing must be tightly regulated to avoid accumulation of potentially immunostimulatory self-RNA and to prevent inappropriate innate immune activation. The SKIV2L RNA exosome has been shown to degrade immunostimulatory self-RNA arising as a cleavage product of the endonuclease IRE-1 during endoplasmic reticulum stress (10), thereby limiting type I IFN–dependent immune activation. However, the role of the SKIV2L RNA exosome for immune homeostasis in the absence of ER stress remains poorly understood.

## RNA stress due to SKIV2L deficiency activates mTORC1 signaling

Loss-of-function mutations in *SKIV2L* cause trichohepatoenteric syndrome (THES2), a rare inborn error of immunity characterized by intrauterine growth retardation, early-onset chronic diarrhea, brittle hair with trichorrhexis nodosa, skin lesions, and immunodeficiency (11, 12). In this issue of the *JCI*, Yang et al. (13) set out to dissect the functional consequences of Skiv2l deficiency in mice. To bypass embryonic lethality of mice with complete *Skiv2l* knockout, they turned to mice with tamoxifen-inducible whole-body deletion of *Skiv2l*. These mice developed inflammatory skin lesions due to impaired epidermal stratification with loss of epidermal barrier integrity. Skin-specific deletion of *Skiv2l* also led to epidermal hyperplasia with defective hair morphogenesis, recapitulating the human disease phenotype. Interestingly, these phenotypic changes were not accompanied by activation of the type I IFN axis, as shown by a lack of expression of IFN-stimulated genes in Skiv2l-deficient epidermis or primary keratinocytes. Moreover, mice with myeloid-specific *Skiv2l* knockout did not show any skin pathology or signs of inflammation, suggesting that skin pathology in *Skiv2l* deficiency occurs independent of the hematopoietic system. Together, these findings indicate a cell-intrinsic mechanism by which the SKI-associated RNA exosome regulates keratinocyte function and which is required for skin barrier integrity independent of type I IFN signaling.

Transcriptional profiling of epidermal tissue of mice with keratinocyte-specific *Skiv2l* deletion revealed enrichment of genes acting in the mTORC1 signaling pathway. The mTORC1 complex, a multiprotein assembly consisting of the serine/threonine protein kinase mTOR, raptor, mLST8, PRAS40, and DEPTOR, acts as a central hub that integrates nutrient signaling pathways to promote cell growth (14). The authors confirmed activation of mTORC1 signaling in Skiv2l-deficient keratinocytes by demonstrating enhanced phosphorylation of ribosomal S6 kinase (S6K) and 4E-BP1, the key downstream effectors of the mTORC1 pathway (14), along with increased global protein synthesis. Skiv2l-deficient skin lesions showed T cell infiltrates and these T cells were chronically activated as shown by increased proliferation in response to ex vivo stimulation with anti-CD3/CD28. Similar to keratinocytes, T cell hyperactivation was accompanied by increased phosphorylation of S6K and 4E-BP1, consistent with an mTORC1-dependent, cell-intrinsic role for Skiv2l in T cell homeostasis. Interestingly, increased epidermal phospho-S6K staining was also observed in lesional skin of a patient with THES2 who presented with failure to thrive, diarrhea, trichorrhexis nodosa, erythematous rash, elevated liver enzymes, and glomerulonephritis, indicating activation of the mTORC1 pathway also in human SKIV2L deficiency. Finally, the authors demonstrated that both systemic and topical treatment of *Skiv2l*-deficient mice with the mTOR inhibitor rapamycin ameliorated epidermal hyperplasia and skin inflammation.

## Conclusions and implications

The mTORC1 pathway promotes cell growth through activation of anabolic processes, including the biosynthesis of proteins, lipids, and nucleotides, as well as through cell-cycle acceleration (14). Thus, enhanced mTORC1 signaling observed in keratinocytes and T cells of Skiv2l-deficient mice provides a mechanistic explanation for uncontrolled hyperproliferation, which is initiated cell-autonomously, resulting in the loss of skin barrier integrity and T cell homeostasis (Figure 1). The T cell phenotype described in Skiv2l-deficient mice is intriguing, because some patients with THES2 develop reactive hemophagocytic syndrome, a hyperinflammatory state caused by hyperactivation of T cells and macrophages (12, 15). Moreover, the findings by Yang et al. indicate that a lack of cytoplasmic RNA quality control due to dysfunction of the SKI-associated RNA exosome is sensed by mTORC1, although the exact nature of the metabolites that are actually sensed by mTORC1 under these circumstances is still unknown.

Cell growth and proliferation requires increased DNA replication, which depends on a sufficient supply of nucleotides (deoxyribonucleotides), the building blocks of DNA synthesis (14). An increased demand for nucleotides is also controlled downstream of mTORC1 through stimulation of de novo nucleotide biosynthesis (16–19). However, the major pathway for biosynthesis of DNA precursors is mediated by ribonucleotide reductase, which generates deoxyribonucleotides from ribonucleotides (20), the end product of the RNA exosome. Thus, while RNA degradation is an inherent step in RNA quality control mechanisms, it also contributes to the recycling of the nucleotide pool in the cell (Figure 1). However, whether changes in cellular nucleotide concentrations underlie mTORC1 signaling in SKIV2L deficiency remains to be investigated.

Interestingly, the authors describe a lack of type I IFN activation in skin and blood of Skiv2l-deficient mice, arguing against a pathogenetic role of RIG-I–dependent innate immune activation by unprocessed self-RNA in SKIV2L deficiency. This result contrasts with work by Eckard et al., which demonstrates an IFN signature in the blood of two patients with THES2, but not in three patients with THES1 who carried mutations in the SKI complex component TTC37 (10, 21), despite having indistinguishable clinical features. The findings by Yang et al. (13) are also in line with a clinical report, demonstrating absence of an IFN signature in a patient with THES2 (15). Nonetheless, a moderately increased expression of MX1, an IFN-regulated gene, was found in skin lesions of the patient studied by Yang et al. (13). The type I IFN activation observed in patients with THES2 might act as a permissive factor rather than as the primary cause of inflammation. This notion is also supported by the therapeutic efficacy of rapamycin in Skiv2l-deficient mice. Patients with THES develop intractable diarrhea commonly leading to failure to thrive (11, 12). Although mice with Skiv2l deficiency do not exhibit intestinal symptoms, it is possible that a loss of intestinal barrier integrity due to aberrant mTORC1 signaling may account for intestinal dysfunction in patients with THES2. As such, rapamycin may provide a promising and potentially curative therapy for these patients.

Perturbations of the mTORC1 pathway have been implicated in a variety of human diseases, including common autoimmune diseases such as systemic lupus erythematosus (22). Given the genetic and phenotypic heterogeneity of these complex diseases, mTORC1 hyperactivation may represent a useful endotype, enabling further stratification of patients based on mechanistic insight.

## Version 1 01/18/2022

Electronic publication
